# Comparisons between sinus rhythm and paced maps using the ‘one acquisition-two maps’ technique

**DOI:** 10.1093/europace/euaf052

**Published:** 2025-05-09

**Authors:** Deborah Foltran, Maxime Beneyto, Jean Timnou Bekouti, Quentin Voglimacci-Stephanopoli, Hubert Delasnerie, Maud Tabuteau, Philippe Maury

**Affiliations:** Department of Cardiology, University Hospital Rangueil, avenue Pr J Poulhès, 31400 Toulouse, France; Department of Cardiology, University Hospital Rangueil, avenue Pr J Poulhès, 31400 Toulouse, France; I2MC, INSERM, University Hospital Rangueil, 1 av. Pr. Poulhès, 31400 Toulouse, France; Department of Cardiology, University Hospital Rangueil, avenue Pr J Poulhès, 31400 Toulouse, France; Clinique Pasteur, 45 av. Lombez, 31076 Toulouse, France; Department of Cardiology, University Hospital Rangueil, avenue Pr J Poulhès, 31400 Toulouse, France; Boston Scientific, 2 rue Caudron, 78960 Voisins-le-Bretonneux, France; Department of Cardiology, University Hospital Rangueil, avenue Pr J Poulhès, 31400 Toulouse, France; I2MC, INSERM, University Hospital Rangueil, 1 av. Pr. Poulhès, 31400 Toulouse, France

**Keywords:** Ventricular tachycardia, Ablation, Substrate mapping, Voltage mapping

## Introduction

During substrate mapping, scar and areas of late abnormal ventricular activations (LAVAs) are usually determined during sinus rhythm or ventricular pacing, but may significantly differ according to the activation wavefront^1,2^. A relevant proportion of abnormal areas may even be missed when mapping is done from only one direction of activation,^3^ thus, maps should be optimally acquired during normal conduction and ventricular pacing. This is rarely done because acquisition of multiple maps is time-consuming. Moreover, catheter locations may diverge even with careful remapping.

We described a technique allowing quicker and reliable comparisons between sinus rhythm and paced maps using a single acquisition, allowing shorter mapping time and precise comparisons of electrograms (EGMs) collected at the exactly same location during different activation wavefronts.^4^ The aim of this study was to precisely quantify the changes in scars and abnormal areas and to try to find some rules about the optimal cardiac rhythm for mapping according to the scar location.

## Methods

Consecutive patients with ischaemic heart disease referred for VT ablation and undergoing the ‘one acquisition-two maps’ technique were retrospectively included.

The protocol has been described in detail elsewhere.^4^ Briefly, a quadripolar catheter was placed at the right ventricular apex (reference/pacing). The external stimulator was specifically programmed to achieve a stable and regular paced ventricular bigeminy during spontaneous rhythm (see Voglimacci-Stephanopoli *et al*.^4^). Left ventricular (LV) endocardial mapping was performed using the Rhythmia™ and Orion™ catheter,^5^ with specific beat acceptance criteria (see Voglimacci-Stephanopoli *et al*.^4^). A first map was acquired targeting the spontaneous QRS complex. Then, the mapping window was moved to the following paced beat and a second map was built in a few seconds, depicting activation and voltage of the paced cardiac beat.

Number of beats was the same in both maps, with exactly the same location of the mapping catheter for each beat. A small difference in the number of EGMs was expected however, but not relevant when looking to the scars.^4^

## Data analysis

Scars were defined by voltage < 0.2 mV, border zone between 0.2 and 0.8 mV and healthy tissues > 0.8 mV.^5^ Numerical data were retrieved in MathLab™ format, and scar areas were automatically calculated using Octave™. Lumipoint™ algorithm was used to highlight the areas of fragmented (threshold seven peaks) or late potentials (confidence threshold 50%).^5^ Highlighted areas were manually measured with the surface area tool.

This study is covered by reference methodology of the French National Commission for Informatics and Liberties (CNIL) and is registered at the Toulouse University Hospital (RnIPH 2024-131).

## Results

Thirty novel patients were included [29 men, 60 years old (17)]. Duration of voltage mapping was 30 min (19). Median LV volume was 163 mL (89) and 154 cm^2^ (65) for LV surface. A median of 609 (325) beats was included in each map, corresponding to 9660 (4785) EGMs in the sinus map vs. 8952 (4898) in the paced map (*P* = 0.003).

A median of one scar (from 1 to 4) was noted in sinus map vs. 1 (from 0 to 3) for the paced map (*P* = 0.4). Locations of the main scar for each patient during sinus rhythm were septal (*n* = 8), apical (*n* = 7), inferior (*n* = 7), infero-lateral (*n* = 5), lateral (*n* = 2), and supero-latero-apical (*n* = 1).

**Figure 1:**
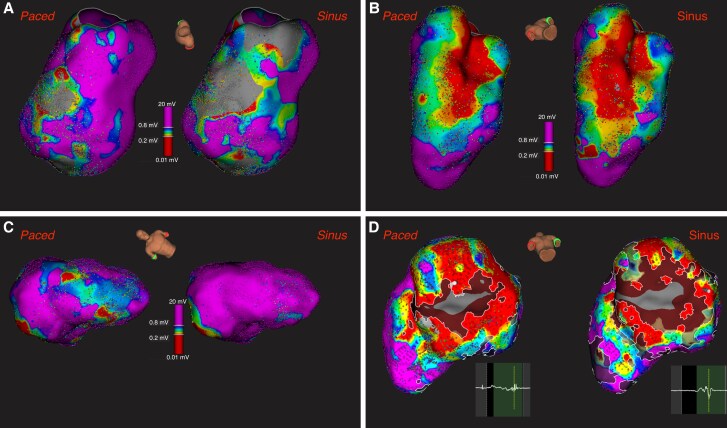
Examples of comparisons of sinus vs. paced voltage maps. (*A* and *C*) Greater septal scar during sinus rhythm (*A*) or pacing (*C*). (*B*) Lack of visual significant difference in inferior scar between sinus and pacing. (*D*) Greater area of fragmented potentials during pacing in an inferior scar.

Differences in scars, late potential, and fragmented potential areas between sinus and paced maps were depicted in *Table 1* and in *Figure 1*.

**Table 1:** Differences in scar, late potential, and fragmented potential areas between sinus and paced maps (median and interquartile range)

	Sinus rhythm map	Paced map	*P*
Healthy surface (>0.8 mV)	68 cm^2^ (23)	94 cm^2^ (26)	<0.0001
Border zone surface (0.2–0.8 mV)	70 cm^2^ (32)	52 cm^2^ (36)	<0.0001
Dense scar surface (<0.2 mV)	14 cm^2^ (30)	12 cm^2^ (20)	<0.0001
Late potential area	4 cm^2^ (9)	2.5 cm^2^ (9)	0.03
Fragmented potential area	9 cm^2^ (15)	4 cm^2^ (9)	0.003
Ratio healthy surface/total surface	42% (18)	57% (25)	<0.0001
Ratio border zone surface/total surface	43% (11)	32% (16)	<0.0001
Ratio dense scar surface/total surface	9.5% (13)	8.5% (12)	<0.0001
Ratio LP area/total surface	2% (0.5)	1.5% (5)	0.03
Ratio fragmented pot area/total surface	4% (1)	2.5% (5)	0.002

Border zone areas were of significant lesser extent in paced maps (averaged difference 18 cm^2^, i.e. 25% less) as were dense scar areas (6 cm^2^, i.e.25% less), leading to a higher area of healthy surface (23 cm^2^, i.e. 25% more) in paced maps. Some scar fully disappeared in paced maps in nine patients (three septal, two apical, two lateral, two inferior).

Areas of late potentials were significantly smaller in paced maps (averaged difference 2.1 cm^2^, i.e. 28% less) as were areas of fragmented potentials (3.6 cm^2^, i.e. 65% less). Late potential areas completely disappeared in four cases on paced maps, and fragmented potential areas in three.

## Discussion

Mapping techniques for VT have been recently summarized.^6^ In this study, we precisely quantified the changes in scars/abnormal areas in ischaemic patients with VT investigated with our ‘one acquisition-two maps’ technique.^4^

Our findings were as follows: (i) border zone and dense scar areas were of significant lesser extent during RV pacing (25% less compared to sinus rhythm), leading to larger healthy surfaces and smaller scar areas in paced maps in the majority; (ii) scars completely disappeared in a third of patients during RV pacing, whatever the scar location; and (iii) areas of late potentials or fragmented potentials were significantly smaller in paced maps, disappearing in some when changing activation. Thus, even if no specific rule about the optimal cardiac rhythm for mapping can be drawn regarding the scar location, voltage maps built during sinus rhythm seem to be more informative in most cases.

A few previous studies had already demonstrated relevant changes in scars and culprit areas while changing the pacing site, but no clear conclusion could be drawn from these works. Using 4 mm tip ablation catheters, a 22% (bipolar) and 14% (unipolar) variability in scar area was noted when comparing different wavefronts of activation, which were generally larger during atrial vs. RV pacing.^2^ Using bipolar high-density mapping, scars were larger and LAVA areas smaller with atrial than RV pacing.^1^ These results were rather close to what we observed.

Although larger scar/LAVAs/fragmented areas were seen in sinus rhythm maps in most cases, no specific rule about the optimal cardiac rhythm for mapping regarding the scar location can be drawn from our work. Larger investigations are required to determine the optimal pacing site in a given patient based on scar location.

It was surprising to observe smaller areas of late potentials after the paced beats, since extrastimuli may reveal concealed late potentials.^7^ However, our results were based on automated reliable detection and calculations. The pacing site and/or the long 400 ms coupling interval may have hinder revelation of concealed LAVAs.

## Authors’ contributions

All authors have contributed to the manuscript.

## Data Availability

Data available on demand.
